# Description of Main Innovative and Alternative Methodologies for Mathematical Learning of Written Algorithms in Primary Education

**DOI:** 10.3389/fpsyg.2022.913536

**Published:** 2022-07-07

**Authors:** María del Carmen Canto López, Malena Manchado Porras, José Carlos Piñero Charlo, Carlos Mera Cantillo, Cándida Delgado Casas, Estíbaliz Aragón Mendizábal, Manuel Antonio García Sedeño

**Affiliations:** ^1^Department of Psychology, Faculty of Science Education, University of Cádiz, Cádiz, Spain; ^2^Department of Didactics of Mathematics, Faculty of Science Education, University of Cádiz, Cádiz, Spain

**Keywords:** mathematical learning, written calculation, Singapore method, ABN method, alternative methods, mental calculation

## Abstract

The traditional way of teaching mathematics generates significant learning difficulties in students that are reflected in their academic performance. In recent years, the number of teachers and researchers interested in finding innovative, flexible and comprehensible methodologies has increased. The main objective of this article has been to carry out a theoretical review of the methodologies for learning/teaching mathematics at school level. Central elements of international and national method initiatives have been highlighted. The empirical evidence on the Singapore method and the Algorithm Based on Numbers (ABN) method confirms the need to include innovative and manipulative strategies in the classroom. The Singapore method has been shown as a source that improves students′ problem solving skill, mathematical competence, boosting reasoning, and a higher motivation. Some studies focused on the effectiveness of the ABN method and its influence on mathematical cognition. The use of this methodology and learning in flipped classroom have obtained significant results in teacher training. These results could provide guidance about how to improve pre-service education in Primary Bachelor’s Degree. The findings presented in the manuscript could be a basis for opening new lines of quantitative research, with the aim of analysing problem solving and the use of manipulative materials in mathematics. Thus, future research should focus on analysing the cognitive processes involved in mathematical learning, carrying out empirical studies in schools. In addition, it is necessary to improve future teachers’ training, so that they can learn about new alternatives for mathematical teaching and the available resources to be able to put it into practice. Moreover, alternative methodologies are a necessary driver for the improvement of mathematical performance both inside and outside the classroom, and also for the technological and economic development of countries.

## Introduction

In recent years, the teaching of mathematics has generated several studies, highlighting the need for a methodological change to reduce the difficulties presented by students. Likewise, the legislative reform in 2022, adapt the official curriculum of Spanish compulsory education to a teaching and learning procedure based on development of mathematical skills (Royal Act 157/2022, 2022). By reviewing the current regulation, Royal Act 157/2022, 2022, of March 1, establishes the organisation and minimum teachings of Primary Education. This document states that “the area must be approached experientially, granting special relevance to manipulation, especially in the first levels, and progressively promoting the continuous use of digital resources, proposing to students learning situations that encourage reflection, reasoning, the establishment of connections, communication and representation. Active methodologies are especially suitable in a competency approach, since they allow knowledge progressive building and the activity of the classroom to be stimulated through the exchange of ideas. Learning situations facilitate interdisciplinary and stimulate reflection, criticism, the elaboration of hypotheses and the research task” (p. 24487).

The legislation recommends the use of active and flexible methodologies for the improvement of mathematical competence. However, daily school practices are generally quite different, because of a type of learning focused on the mechanisation of calculus. In the end, the development of mathematical competence is limited to developing the ability to do calculations by using a non-flexible algorithm and makes an extensive use of decontextualized written algorithms.

**FIGURE 1:**
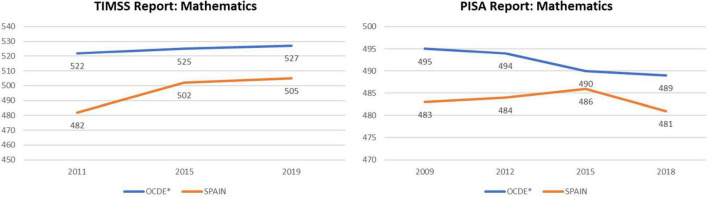
Progress of the mean scores in mathematics from Trends in International Mathematics and Science Study report (TIMSS) and Program for international student assessment report (PISA). *OECD, Organisation for Economic Cooperation and Development.

To describe the current situation of the teaching of school mathematics in Spain, it is convenient to analyse the scores obtained in the two main sources of information. One of them is the State System of Education Indicators (SEIE), which collects data on the results of national diagnostic evaluations for the years 2011, 2015, and 2019. The second shows the results of schoolchildren in evaluation tests carried out by international organisations in 2009, 2012, 2015, and 2018. In both cases the data were obtained from each of the two compulsory stages of the Spanish education system: Primary Education (from 6 to 12 years) and Secondary Education (from 12 to 16 years). Figure 1 shows the evolution of the mathematics performance of Spanish students with respect to the values of the OECD countries. Furthermore, the results obtained in the TIMSS assessment (2019) showed a slight no significant decrease in 2019, compared to 2015 report. However, it should be noted that widens the gap with the OECD average, from 20 points in 2015 to 25 in 2019. This is, in any case, far from the 40 points that occurred in 2011. On the other hand, the results of the latest PISA international report (2018) showed that the scores obtained by Spanish students continued below that of previous evaluations. The results’ progression presented on these reports raises the urgency of a methodological innovation in the area of mathematics. Such an innovation should promote reasoning, mental calculation and the use of flexible algorithms which adapt to the specific differences of the students, with the aim of improving their mathematical skills.

Analysing the mathematics’ learning process in schools, it is found that it is perceived as a topic that has shown no meaningful evolution in its teaching method. That is, the problems that arise from learning basic operations have existed for decades, so after the generalisation of the use of calculator devices, the teaching of traditional algorithms and their function in school began to be questioned (Barba and Calvo, 2012).

As a consequence, more and more teachers are looking for an alternative method to teach mathematics (specifically, a method focused on contextualising routines and generating strategies to ease the acquisition of mathematical knowledge). They are aware of the difficulties that the traditional way of teaching generates in the students and they require training support to implement these needs for change.

Currently, the controversy on the use of traditional algorithms and the methodology used for learning mathematics is a significant issue in the Spanish educational area (where many teachers are interested in learning new strategies). These teachers need training support to carry out a meaningful renewal in mathematical teaching with a guarantee of success. From this situation, interest arises in researching and analysing new methods of learning written and mental calculations, which are more open, flexible, and understandable. A type of mathematics that is applicable to everyday life problem solving. In the present work, a review of some of the methods of teaching mathematics that are applied in the classroom and affect other strategies to teach mathematical content were analysed. These strategies tend to be more based on the development of number sense, decomposition strategies and mental calculation for maths operations, the use of the calculator-device and problem solving. These methodologies try to encourage students to invent and develop significant calculation strategies, both written and mental, for reasoning and understanding operations and quantities.

## Innovative Mathematical Teaching Practices

The aim of this section is to deliver a synthesis of the main practises on the field of mathematics teaching. That is, the results are intended to provide an overview about the different alternative mathematics teaching methods that exist today and that have sufficient empirical evidence. Thus, this manuscript describes the characteristics of some innovative methodologies, so that the reader can learn about them and judge their strengths and weaknesses.

A few years ago, alternative strategies for learning mathematics began to be used in different countries. Considering the universal references on mathematics education and the current regulatory frameworks in developed countries, the importance of promoting schoolchildren’s “number sense” and mental calculation strategies. Such skills are, indeed, oriented to the development of the mathematics competence (García et al., 2011). Some authors highlight the influence of the use of alternative and innovative strategies in technological and economic development (**Aguilar et al., 2015**; **Passolunghi and Costa, 2016**). Even though the referents agree on the need for a methodological change in the learning of calculus in Early Childhood Education and Primary Education, in the classrooms’ daily life, calculus continues to be taught through the traditional algorithm (CBC, by its Spanish acronym). CBC algorithm uses repetitive and mechanical learning as the basis of the methodology. As a consequence, low motivation in students toward learning mathematics is observed. This low motivation is extended to a section of the teaching staff when referring to facing innovation in this matter; methodological renewal initiatives are being carried out with direct practical effects in the classrooms, focused on the need to improve mathematical competence. For this reason, the main objective of this paper is to carry out a theoretical overview of the teaching-learning methods of school mathematics, presenting those with the highest empirical contrast and focusing on the “alternative algorithms and innovative methodologies” such as the **Algorithm Based on Numbers** (ABN) method, because is included in one of the lines of work of the research group.

### The Netherlands Algorithms

The Netherlands bases mathematical education on the so-called Realistic Mathematical Education (RME) (Van den Heuvel-Panhuizen and Drijvers, 2020). Freudenthal (1968), defined RME as a learning philosophy that implies the following main ideas: (1) mathematics is a human activity and therefore must be accessible to all people, it is an activity in constant transformation; (2) their teaching must be connected to the reality and experiences of the students; (3) The development of mathematical understanding goes through different phases progressively, (4) The models that guide the learners have a relevant role in said development; (5) finally, the learning process of mathematics is a process of guided reinvention, that is, the model must provide students with contexts or situations that lead to the need to apply mathematical concepts and generate spontaneous mathematical productions. In this way, maths sessions in the classroom should be an opportunity to reinvent maths by “doing it.” Therefore, to apply this to the classroom, it is necessary to have two basic resources: models or mediators between the concrete and the abstract issues, and the interaction in the classroom between students and teacher, which allows him to adapt his classes’ content to the students’ results. In short, from this perspective, mathematics education constantly seeks for students to develop their own cognitive strategies for solving problems.

The application of the Dutch teaching methodology is implemented in the classroom through mental calculation, column calculation and alternative algorithms for the basic operations of addition, subtraction, multiplication, and division (Van den Heuvel-Panhuizen and Wijers, 2016). Mental calculation in the Netherlands is considered a basic element in Primary Education, and the most common approaches are made by chaining, splitting and variations (varying). For example, the operation 253 + 198 could be approached in three ways: chaining, separation, and variations. These strategies are presented in Table 1. Column calculation is an alternative to algorithmic calculation, standing at a midpoint between it and mental calculation. In this regard, it is possible to use the strategies mentioned for mental calculation. Column calculation is done by writing vertically, from left to right, as in the traditional algorithm. So, the student works with entities that have meaning for them, and not with abstract symbols that are handled by an inflexible algorithm mechanically learned.

**TABLE 1:** Strategies for mental calculation used in The Netherlands algorithms.

(1) Chaining	253 + 100 = 353; 353 + 90 = 443; 443 + 8 = 451.
(2) Separation	200 + 100 = 300; 50 + 90 = 140; 3 + 8 = 11; 300 + 140 + 11 = 451
(3) Variations	253 + 200 = 453; 453 − 2 = 451

*Three ways to solve an addition.*

**FIGURE 2:**
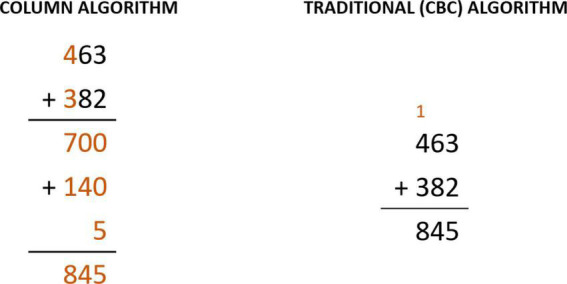
Forms of calculation vertically in traditional algorithms.

Finally, as an alternative algorithm for the basic operations of addition and subtraction, the decomposition system is used. Once its domain is reached, students transition to the traditional algorithm. As an example, the column algorithm highlighted in Figure 2 shows a decomposition strategy for addition. As for multiplication, decomposition is again used as a bridge to manage the traditional algorithm. Decomposition involves starting with the hundreds, continuing with tens, and ending with the ones. For example, 365 × 7 would be solved as is presented in Table 2. Finally, the division involves an additional level of complexity, for which special interest is given to the adaptation to the learning rhythm of each student. Thus, a division can be performed in as many steps as necessary, 420 ÷ 12 could be solved as follows (see Table 3).

**TABLE 2:** Example for multiplication using decomposition strategies.

365 × 7	300 × 7 = 2100; 60 × 7 = 420; 5 × 7 = 35; 2100 + 420 + 35 = **2555**.

*The Netherlands algorithms. Bold values significate are the calculation results.*

**TABLE 3:** Example for division using decomposition.

420:12	12 × 10 = 120; 420 − 120 = 300; 12 × 10 = 120; 300 − 120 = 180; 12 × 10 = 120; 180 − 120 = 60; 12 × 5 = 60; 60 − 60 = 0; 10 + 10 + 10 + 5 = **35**.

*The Netherlands algorithms. Bold values significate are the calculation results.*

### American Algorithms

There really is no American algorithm itself. However, there are many studies reviewing the effectiveness of the use of the traditional algorithm and aims to leave it to the student to invent their own algorithm, in the way that seems simplest and according to their thinking. As indicated by Pérez et al. (2018), traditional algorithms do not encourage students’ autonomous thinking, since they are based on memory processes and certain predetermined steps. In this way, students are able to operate without understanding the concept or the place value of numbers, thus causing them to lose number sense. Based on this argument, student-designed algorithms are encouraged, since the use of these algorithms provides a freedom of thought from students, they proceed to universally operate from left to right (unlike the CBC algorithm) (Madell, 1985; cited in Fuson, 2020). Therefore, the traditional algorithm forces schoolchildren to treat each number column as if it were a unit’s column and not make evident the whole number.

The work on alternative algorithms in the United States is based on the fact that it is the students themselves, by facing a problem, who apply the strategies that are most suitable for them to solve it. As well as that they share their ideas with other students, so that feedback is generated from students and the classmates learn. This process occurs with the teacher’s help. He/she will guide the student in developing their own resolution strategies.

#### Addition and Subtraction

**FIGURE 3:**
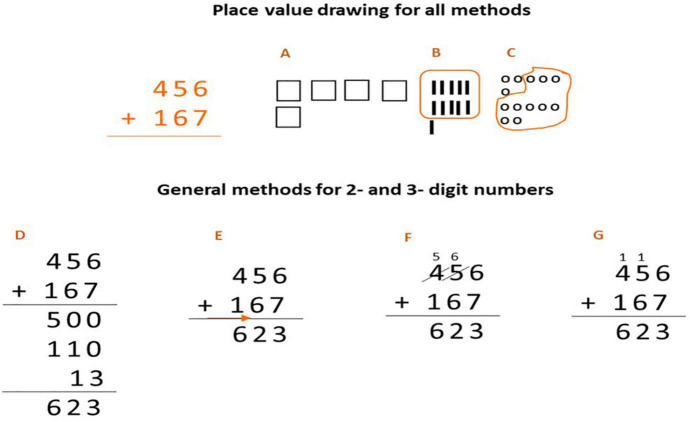
Alternative addition algorithms used in the United States’s education system. Source: Fuson and Beckmann (2013, p. 19). Methods (A,B) variations of what is counted or added on first are possible, and the number of steps involved could vary. Method (C) keeps track numerically rather than with a drawing. Methods (D–G) are all variations in the standard algorithm, but Method (D) is conceptually clearer and easier.

Here is a counting or adding approach that starts with an unbroken number. For the resolution of the sum (Figure 3), several variations that exist for a sum of two three-digit addends are presented: 456 + 167. In formats A, B and C, a visual support is used to carry out the sum; while models D, E, F and G do not have visual support but work directly with numerical signs, making less and less use of decomposition.

**FIGURE 4:**
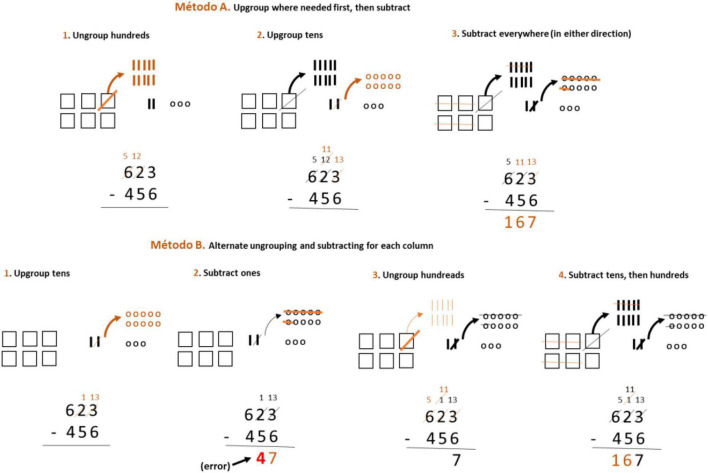
Alternative subtraction algorithms used in the United States’s education system. Source: Fuson and Beckmann (2013, p. 21).

Fuson and Beckmann (2013) presented an alternative format for three-digit subtraction. In this case, two different methods are suggested: Method A, first ungroup when needed, then subtract. This method allows the procedure to be carried out from left to right and from right to left; And method B, alternates ungrouping and subtracting in each column (Figure 4).

#### Multiplication and Division

**FIGURE 5:**
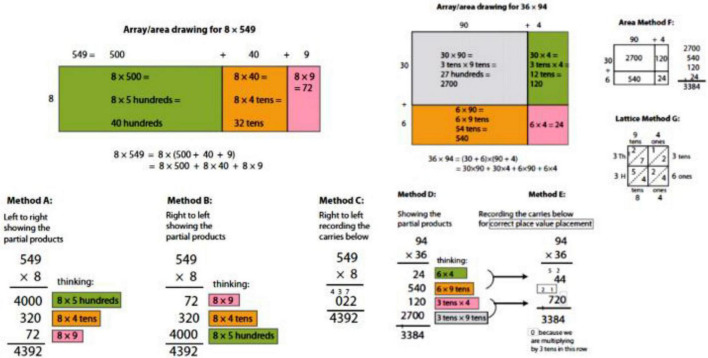
Alternative multiplication and division algorithms used in the United States’s education system. Source: Fuson and Beckmann (2013, p. 23).

Three different methods (A, B and C) are proposed for the multiplication format of 3 digits times 1 digit. It is based on the representation of the product from the decomposition and positional values. In method A, it is carried out from left to right, and the others from right to left. In Method C, the digits representing new units are written below the line rather than above the multiplicand, thus keeping the product digits close to each other. For example, in 8 × 9 = 72, the digit 7 is written diagonally to the left of the 2 instead of writing it above the digit 4 in 549 (Figure 5).

Figure 5 also illustrates the two methods proposed by Fuson and Beckmann (2013) for the 3-digit by 1-digit division algorithm. In the first place, method A includes both the memory of the numerical calculations from the distributive property with respect to addition, and the area model that corresponds to the calculations. In method A, the total multiplier of the divisor at each step is written above the dividend so that students can see the place value and make a clear connection to the place values in the area model. The full product is written at each step, and the unused dividend amount is also written in full.

After the experience of connecting to a graphical representation, method A can also be carried out without such a representation, as a traditional algorithm for calculating quotients that are whole numbers or decimals (method B).

### Methodologies and Learning Alternatives at the National Level

#### Other Algorithms for Arithmetic Operations Method

An additional example of an alternative methodology for teaching mathematics is the OAOA approach. This methodology opposes traditional algorithms, promoting flexible and diverse algorithms. This implies the understanding that abstract mathematical symbols have a specific meaning. Furthermore, this alternative defends the use of the calculator-device in the classroom, arguing that it is a useful tool to investigate the properties of numbers by working with them, at least from 4 years of age, as well as to learn multiplication tables. In this way, when facing a calculation, the students would be prepared first, to make an estimation or an approximate calculation, and then use the calculator. In other words, the resolution of the operation would be covered first with a mental process, and then with a digital process. To achieve mastery, it is essential to train mental calculation capacity, which, on the other hand, is a fundamental skill for any mathematical operation in daily life (Iglesias and Martín-Adrián, 2011; cited in Bracho-López et al., 2014).

It is proposed that the process of learning mathematical concepts follows three phases, based on Bruner (1960), and reminiscent of the CPA (Concrete, Pictorial and Abstract) approach of the Singapore method. (1) Manipulative phase: it consists of using manipulative materials to understand the mathematical operations that must be carried out, expressing the process orally. (2) Graphic phase: it is about representing the process previously carried out with the manipulative materials through drawings, diagrams, etc. (3) Symbolic phase: written text and numerical symbols are included. This author argues that, at least in part, school failure in mathematics is due to a breach of the order of the learning phases, since in traditional classrooms, the first two phases are left in the background, focusing the sessions on paper and pencil activities that directly involve abstract mathematical symbols (Putri, 2015).

Finally, the OAOA methodology emphasises that an adequate handling of the concepts of units, tens, hundreds, etc., and the relationships between them, is essential before starting the learning process of the algorithms to solve the basic operations. These basic operations would be worked after the basic concepts, through strategies such as the decomposition of numbers that make up an addition, subtraction, multiplication or division. Two clear examples would be the tree format or the spider format. The first is to break down the dividend in a way that makes it easier to do the operation mentally. For example, if we want to divide 485 by 5, we break 485 into 450 and 35. 450 by 5 gives 90, and 35 by 5 gives 7. Therefore, the desired result would be 90 + 7 = 97. The second consists of breaking down the dividend in this case into hundreds, tens, and ones, divide each of those components and add the results. For example, if we want to divide 485 by 5, we will divide 400 by 5 (80), 80 by 5 (16) and 5 by 5 (1), and the result would be 80 + 16 + 1 = 97 (Iglesias and Martín-Adrián, 2011; cited in Bracho-López et al., 2014).

#### Singapore Method

The learning that occurs in the classroom should not be a passive act or isolated from reality Opposite, students should be able to apply knowledge to solve real problems on their context, since the problem-solving process is present in the daily life of people, in any of its spheres (Riscanevo-Espitia, 2016; Bernate et al., 2020). Applied to mathematics, problem solving must be led by reasoning, that is, it must be based on the logical search for procedures, a deep understanding of concepts and the explanation and justification of each step, without giving rise to arbitrariness (Niño-Vega et al., 2020).

In recent decades, Singapore has become a world economic power that has a solid educational system, focused on promoting autonomous learning. Such a learning methodology aims to allow students to explore various strategies and build their own knowledge, which moves them away from purely rote learning (Zapatera, 2020). Thus, the Singapore method has a history of more than 30 years of application in the classrooms of that country, and has expanded to the United States, Colombia, Chile and Spain (Juárez and Aguilar, 2018). Its effectiveness has been shown in the international assessments results (Organisation for Economic Co-operation and Development Organisation for Economic Co-operation and Development [OECD], 2016, 2019).

The curricular framework of the Singapore method has as a central element the resolution of mathematical problems (Zapatera, 2020). It is considered a challenge that forces students to hypothesise and investigate the underlying mathematical concepts. This ability is developed through five basic elements: attitudes, skills, concepts, processes and metacognition. Attitudes refer to affective-emotional aspects, such as interest, motivation or perseverance in the matter. Maths skills include calculation, spatial reasoning, measurement, estimation, etc. The concepts cover different mathematical areas such as algebra, geometry, statistics, etc. The processes encompass mathematical investigation, reasoning, application and communication procedures. Finally, metacognition refers to the self-regulation of learning and of one’s thought sequence. From the perspective of the Singapore method, all these elements are important when it comes to mathematical learning and, therefore, they must be addressed in the classroom: from the creation of an environment that encourages a positive attitude toward the subject, to the training of the understanding of specific concepts.

The Singapore method focuses attention on the process rather than the result, avoiding routine and rote procedures, and seeking understanding and reasoning, through four fundamental methodological resources: (1) the CPA approach (Concrete-Pictorial-Abstract); (2) the spiral curriculum; (3) systematic and perceptual variations; and (4), relational rather than instrumental understanding (Zapatera, 2020).

**FIGURE 6:**
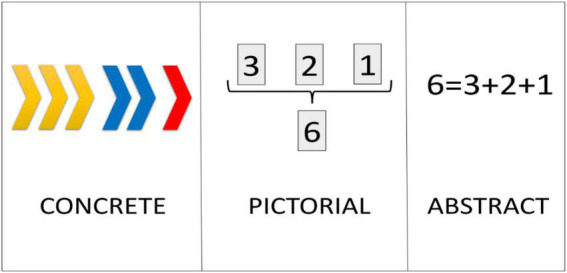
Example of application of the Concrete Pictorial Abstract approach.

The CPA approach is based on the consideration that, in order to achieve true learning, the understanding of concepts must be deep and exhaustive, covering three levels graded by difficulty (Figure 6).

##### Concrete

The concrete level involves working in the classroom with attractive and everyday materials such as chips, cubes, balls, etc., to help students understand a certain mathematical concept.

##### Pictorial

The student is induced to advance in the understanding of the concept, represented in this case with graphic elements such as figures, drawings or images.

##### Abstract

It is the most difficult level. Understanding the concept implies representing it through mathematical symbols or signs, connecting the concept with abstract mathematical algorithms.

The CPA approach derives from representation proposed by Bruner (1960): enactive, iconic, and symbolic, which refer to the representations of concepts through action, images, and symbols, respectively. In addition, Bruner, as a representative of Discovery Learning, defends students should have an active role in the learning process, managing the content themselves, instead of being limited to being passive agents who receive information (Putri, 2015).

The spiral curriculum involves covering content repeatedly and at different levels. In other words, the concepts are worked on repeatedly at different times during the school career, encouraging review and gradually increasing the level of complexity and abstraction. This second methodological resource justifies the progressive and reiterative presentation of the contents based on the progressive nature of cognitive development. The learning process must increase in complexity and adapt to the students.

Systematic and perceptual variation refers to the presentation of the same concept in different ways, with different degrees of complexity and abstraction. In this way, students can acquire the concept in the way that best suits their abilities and interests.

Finally, relational understanding emphasises the relationship between the concepts taught in the classroom and different situations in everyday life, moving away from the instrumental approach focused on memorising rules that apply to very specific situations. The Singapore method differentiates between instrumental understanding (which implies “knowing how to do”) and relational understanding (“knowing why to do it”). The first requires establishing a few steps to follow to reach a result, thus generating a relatively simple and mechanical process. The second requires mastering the concepts so that they allow the creation of different strategies to reach a result.

#### Open Method Based on Numbers

One of the main alternative methodologies for teaching mathematics that are being used in many schools internationally is the so-called ABN method. This methodology has been tested in the classrooms of different countries in the last ten years, calculating that more than 250,000 Spanish-speaking schoolchildren are learning mathematics with the ABN method (Cerda et al., 2018). One of the reasons for its success is the fact that it is a meticulously sequenced and structured method. It offers a logical and clear sequence, from the beginning of the Early Childhood Education stage and up to the third cycle of Primary Education, coming to address mathematical content typical of the first cycle of ESO.

Martínez-Montero and Sánchez-Cortés (2019, 2021) emphasise that the ABN methodology is a method that deals, fundamentally, with calculation, using it for conceptual knowledge. It is a key element for the development of formal thought, and it is an essential part of the mathematical knowledge necessary at school. It should be noted that this methodology facilitates the connection between numbering and operations for problem solving. It stimulates mathematical reasoning and makes it possible for the child to adapt the operations to their own individual characteristics and to their learning pace. In short, this methodology seeks to adapt the method to the students. This translates into the search for various possibilities to complete the same task, solving it according to their abilities.

##### Background of the Algorithm Based on Numbers Method

As antecedents of the ABN method, it is worth noting the alternative calculation procedures advocated by Ramírez and Usón (1996, cited in Cerda Etchepare et al., 2021, p. 45) and the National Strategies *Guidance paper-calculation*, where presented the use of “expanded methods” to support children in written methods, offering a link between mental methods and written algorithms (DfES, 2010, cited in McNeill, 2014, p. 69). It contains the methodological principles of the important renewal of mathematics that is taking place in the United Kingdom. For example, it uses four “stages” for the introduction of the addition: (1) on the number line; (2) starting the partitions of the numbers; (3) using the classic algorithm, but using one row for each numerical combination; and (4), use of the classical vertical algorithm.

The ABN method would also have clearer precedents of various educational actions launched in the Netherlands with the aim of renewing the teaching-learning of calculus. Specifically, we refer to:

*“Design of a national program for mathematics education in primary schools”* (“Proeve”) (Treffers et al., 1989, cited in Gravemeijer et al., 2016). It is a support guide for authors of textbooks, teacher trainers, educational advisors and inspectors, made with a didactic style and plenty of drawings and examples.

*“Sketches of longitudinal trajectories of teaching-learning,”* launched in 1997 to the present. This program collects the steps that the teacher has to carry out so that the students reach the established objectives. It provides teachers with a narrative outline of how the learning process can be carried out, including work materials, examples, videos, etc.

##### Theoretical Bases of the Algorithm Based on Numbers Method

The theoretical foundation of the ABN method is found in Piaget’s constructivist models (Martínez-Montero, 2011; cited in Navarro et al., 2014). Regarding the teaching-learning methodology of mathematics, ABN is based on the “Realistic Mathematics Teaching” (RMT) approach. The goal is to have direct contact mathematics-reality, associating mathematics with children’s experiences and to having a social and human value. The RME approach was created under the ideas of Freudenthal (1968), a mathematician and educator, a promoter of a change in the traditional teaching of mathematics. Freudenthal conceives of mathematics as a human activity that consists of mathematics: organising or structuring reality, including mathematics itself (Zolkower et al., 2020).

##### Characteristics of the Algorithm Based on Numbers Method

The ABN calculation methodology represents a very important change in the quantity and quality of children’s mathematical achievements and requires specific teacher training and a change in mentality about teaching of mathematics. The method can be defined as a calculation and problem-solving method, having some specific characteristics (Table 4). Work on numbering as a basis for mathematical learning and support for calculation and problem solving. It promotes natural and open learning, through which students can solve operations and problems in different ways, considering the individual progress of each student. This is specified in that students can learn faster. The method helps them improve their ability to estimate and mental calculation. The ABN method allows each student to perform the operations according to their own ability, which translates into an effective improvement in motivation and a significant change in the students’ attitude toward mathematics (Martínez-Montero and Sánchez-Cortés, 2021).

**TABLE 4:** Characteristics of Open Algorithm Based on Numbers method.

Characteristics	Significate
1. Based on numbers	• Work with numbers, with complete quantities and not with ciphers (*). • Have a thorough understanding of the numbering system. • Use the left-right direction to perform calculations
2. Open calculation	• The need to adapt to the learning pace of each student • The format allows each student to develop the steps they need, with a great variety of examples of the different types of cognitive processing and learning rhythms in the same classroom
3. Realistic approach	• Based on the resolution of real and contextualized problems • Problems can be formulated and reasoned by the students • Small group work and sharing in the group-class • The teacher acts as a guide for learning
4. Conceptual learning	• Faster and more meaningful learning. • Facilitates the recall of procedures. • Encourages a positive attitude toward mathematics • It is a tool to learn to learn autonomously

(*) The treatment that is carried out in the operations varies substantially in the closed and open learning methods. In closed algorithms, the treatment of quantities is done by separate numbers, based on positional order (as in the abacus); For example, if we add 25 + 18, the sum would be resolved according to its figures: 5 + 8, equal to 13, I take one, so I would add 2 + 1 + 1, which I take. However, in the open methods, full quantities are worked with, accompanied by the representation with manipulative material; in the sum 25 + 18, we would add 25 + 10, which is 35, and then 35 + 5, which is 40, and finally 40 + 3.

##### Algorithms and Mental Calculation of the Algorithm Based on Numbers Method

The algorithms used in the ABN method allow students to mentally implement maths strategies. The first stage in all maths facts is to use manipulative resources.

###### Addition

**FIGURE 7:**
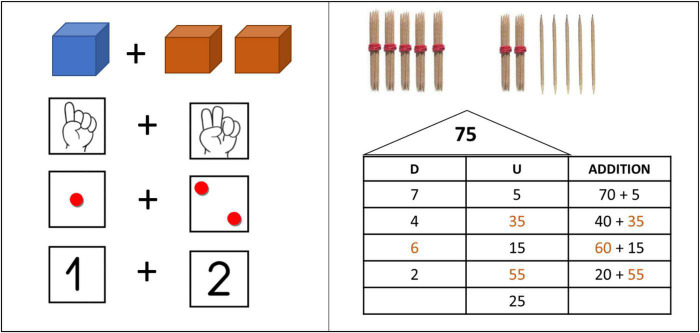
Manipulative resources for teaching mathematics with the Open Algorithm Based on Numbers method.

The essence of addition is to accumulate an added to the other. Once fully accumulated, the new addend provides us the result. In the traditional algorithm, the format can be only done on one way: decomposing the addends into units, tens, hundreds.; placing them properly and, finally, making a unit-to-unit combination and following the order from lesser to higher (no exceptions and no possibility to modify this rule are allowed). At the learning beginning stage, children use different manipulative materials such as toothpicks, fingers, bottle caps, blocks… (Figure 7).

**FIGURE 8:**
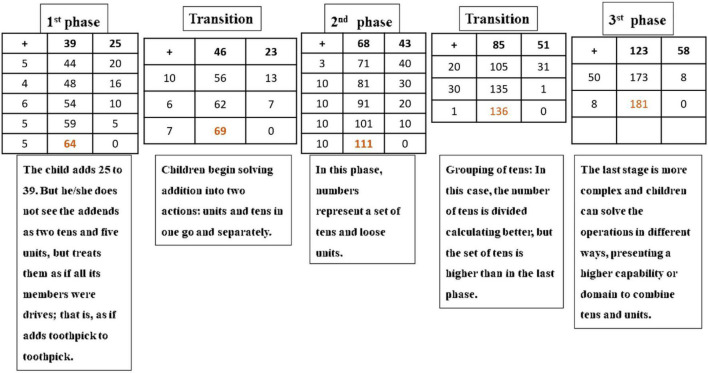
Addition phases in Open Algorithm Based on Numbers method.

Then, when children acquire a basic mental calculation, the writing algorithm can be introduced. Addition has different phases (Figure 8).

###### Subtraction

**FIGURE 9:**
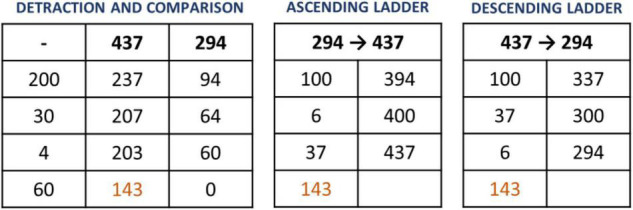
Three subtraction formats in Open Algorithm Based on Numbers method: detraction and comparison, ascending ladder and descending ladder.

In subtraction three different basic models are used (Figure 9), which are adapted to different types of problems (Martínez-Montero, 2011).

*Detraction and comparison format*: This type of subtraction is solved by removing from both terms the same amount until the smallest disappears. The resulting number of the highest amount is the result.

*Ascending Ladder format*: This subtraction process is the most natural and the most likely to be employed by children. It is the system used in the operations of giving change in shops. This format only uses two columns. In the first one, the partial amounts are collocated. In the second one, the progress is represented until the desired amount is obtained. The process is easy to explain.

*Descending ladder format*: The process is opposite to the ascending stair format. The highest amount is changed in the smallest, in two columns. The typical problem model for this format is: How many floors do I have to descend from the 437th floor to the 294th? The goal of this format is to subtract different amounts from the larger amount arriving at the smaller amount, and then adding the subtracted amounts.

###### Multiplication

**FIGURE 10:**
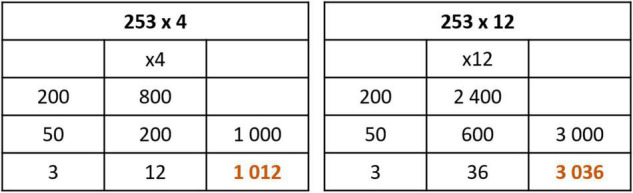
Multiplication algorithms (one digit and two digits) in Open method Based on Numbers method.

There are less multiplication alternatives because it requires tables’ memorization. The process should be: decomposing the factor into units, tens, and hundreds. And then, the partial products are added. Figure 10 shows an example of how some children decompose the numbers in a different way depending on the children’s capacity.

###### Division

**FIGURE 11:**
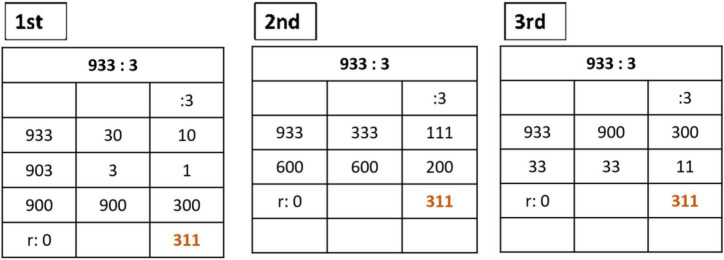
Division algorithm with three columns in Open Algorithm Based on Numbers method.

Division allows different adaptation levels and breakdown of the calculations. In the initiation process, children express different domains depending on their ability with the times-tables. Once children know times-tables and are able to multiply units, tens, hundreds and thousands; then they can solve all divisions by one cipher on the quotient. This algorithm consists of three columns. The first one on the left represents the total amounts to be distributed. The second one, in the centre, represents the amounts taken by the child for doing the accurate distribution. The column on the right represents the partial quotients. The sum of them represents the total quotient and the amount in the first column is the remainder. The next examples show the different ways to solve the divisions, depending on the children’s skills (Figure 11).

Summarizing, the above-mentioned methodologies has been presented by a descriptive review that could serve as a guide for researchers and teachers. For researchers, it could help to identify the mathematics teaching methods that need additional research on their empirical evidence or theoretical bases. For teachers, it could guide them in selecting the methods and strategies to be implemented in the classroom.

## Discussion

Responding to research problems, the aim of this paper is to provide an overview about the different mathematical teaching methodologies most commonly used in Spain. Besides, this paper pretends to describe mathematical learning, some disadvantages in the traditional mathematics teaching methods, and the different emerging teaching maths alternatives approaches in recent years. From this overview, it is clear that the methodology followed for decades in several countries has some limitations to facing students’ mathematical situations, both in and out of the classroom. That is, some traditional methodologies leave in the background such important elements as the understanding of mathematical concepts, the connection of these concepts with real life, the students’ motivation and attitude, the management of mental arithmetic, the diversification of solution strategies, the self-regulation of learning and the adaptation of teaching process to the different learning rhythms. Therefore, innovative methodologies that incorporate these features, and use varied teaching strategies and techniques, as well as resources connected to children’s context (such as manipulative or graphic tools), are an important driving force for the improvement of students’ mathematical performance. Moreover, innovation in mathematics teaching would not only have a positive influence on academic performance, but also on the multiple mathematical daily life situations (Aguilar et al., 2015; Passolunghi and Costa, 2016).

Regarding the empirical evidence, the results of the two most contrasted methodologies used in schools in Spain are highlighted. Considering the empirical evidence of the Singapore method, several studies reflect its advantages in mathematics learning. Espinoza et al. (2016) compared scores in different mathematical skills between fourth grade Primary Education students. The research was composed for two study groups: a treatment group (Singapore methodology, *n* = 459) and a comparison group (Traditional methodology, *n* = 221). The results showed that the treatment group obtained an average achievement percentage 4.76 points higher than the comparison group. The scores were higher in all the skills evaluated, including problem solving, manipulating mathematical expressions, and representing mathematical concepts. A T-test was performed for two independent samples to determine the statistical significance of this difference. The results of this statistical test showed that the difference between the scores in schools implementing the Singapore Method is statistically significant.

Juárez and Aguilar (2018), carried out a study to contribute to the improvement of mathematics learning in Primary Education using the Singapore method for problem solving. The research methodology used was quantitative (pre-test and post-test) and qualitative (participant observation). The design was quasi-experimental and the sample was 31 second-year children from a primary school public of the state of Puebla (Mexico). Results showed that children improved their learning in mathematics after the Singapore method. A total of 70% of students were able to solve maths problems that involved adding or subtracting.

Meneses-Patiño and Ardila (2019), different didactic addition problem solving strategies, based on the Singapore approach, in second and third graders. The qualitative analysis of participants showed that these strategies were useful improving both, the understanding and the achievement of mathematical problems solving. That is, the use of manipulative, pictorial, cooperative and reality-connected resources allowed cognitive processes training. These strategies were used by the students getting a better understanding, an optimal performance and a higher motivation.

In another study conducted by Niño-Vega et al. (2020), studied why students struggle with understanding fractions and they were not able to apply it to a real context (*n* = 35). They carried out a classroom intervention based on three typical activities based on the Singapore method in 8th grade of Primary Education: firstly, concrete manipulation; secondly graphic representation; and finally, the handling of the abstract concept. The results of the study showed that, after the intervention, students achieved better and showed a higher understanding of fractions. They got a significantly higher score in the final test, concluding that the use of the Singapore method facilitates the assimilation of concepts by the student. This is due to the fact that, by initiating the intervention with a concrete object and manipulation, the appropriation of the concepts is made easier by associating them to everyday life.

Another teaching mathematics method that has different empirical studies has been the ABN method. In recent years, some studies have been carried out that contrast the results obtained in mathematics by students who follow a more traditional methodology, compared to others who have learned with the ABN method. Although it is essential that this type of controlled studies be carried out to give scientific validity to the ABN method, there is a considerable number of testimonies, videos, and didactic experiences showing its efficiency. We summarise below some of the evidence carried out.

Martínez-Montero (2011, cited in Aragón et al., 2017a), presents the results of the study carried out during the 2009–2010 academic year. The sample consisted of 99 participants, from 2nd year of Primary Education (7–8 years old) belonging to 4 schools (two public and two private). The experimental group (*n* = 49) had only worked on calculus following the ABN method since the beginning of their schooling; the control group (*n* = 50) had worked through the traditional method (CBC). In the first week of June 2010, the evaluation tests with tasks of mental calculation, resolution of operations and explained resolution of problems were applied. The data obtained showed that the students who followed the ABN methodology obtained better results in the tasks presented, achieving a much higher level of achievement than those who followed the CBC method. Significant differences were obtained in all applied tasks (*p* < 0.001), except for a mental arithmetic product. It was concluded that the ABN method achieved a better performance of the students with lower performance in mathematics.

Bracho-López et al. (2014) analysed the degree of development of number sense reached by a sample composed of 46 students in the second year of Primary Education, after using the ABN methodology (*n* = 20) or traditional calculation algorithms (*n* = 26). After applying the Mathematical Competence Test (TEMA-3), significant differences were found (*t* = 3.028*; p* < 0.004) between the means of the mathematical competence index of both types of methodology. In this same direction was the study by Bracho-López and Adamuz-Povedano (2014), adding the analysis of the TEMA-3 scores obtained by students with special educational needs (NEAE). Special attention was paid to the different learning rhythms in the classroom. Three up to six students with low intellectual functioning and autism spectrum disorder, reached an average and above-average mathematical competence scores.

Canto-López (2017) also found an improvement in mathematical learning with the ABN method, compared to the traditional approach. Data referred both to mathematical performance tasks and cognitive processes involved in mathematical learning (numerical approximation system, processing of numerical magnitudes, and estimation on the mental number line). The sample consisted of 49 students who were in the 4th grade of Primary Education (9-year-old). The students belonged to two public schools in an Andalusian town of 80,000 inhabitants, of a medium socioeconomic level. Both schools worked with two different mathematical learning methodologies, establishing two study groups: control group, which had used a traditional method based on CBC algorithms; and the experimental group, the ABN method. The participants were individually evaluated during the third quarter of the academic year. An *ad hoc* test that was administered to both groups for the evaluation of mathematical knowledge. The test was composed of numbering tasks, written maths-operations, problem solving and mental calculation. Higher means were obtained in the experimental group (ABN), which presented a statistically significant mathematical performance (*p* < 0.001) compared to the CBC group.

Some authors have focused on the study of the ABN methodology with college students. Piñero Charlo and Canto López (2019) presented the implementation of the ABN methodology in the teaching of college students participants during the 2017–2018 academic year. A flipped classroom methodology was used, providing resources in different media to the different work groups (video lessons or text lessons), in order to discriminate the performance of these resources. The sample was made up of 130 students of the Primary Education bachelor’s degree. The mixed group consisted of 56 students (43 women, 13 men). The traditional learning group had a total of 74 students (51 women, 23 men). The majority of students in both groups were in their second university academic year (54 from the mixed group, 67 from the traditional group). Experimental data were collected through individual and group interviews, field notes, individual homework, a questionnaire, and a final exam. The results show a higher performance in the blended learning group than in the traditional group. In this area, recently, Jeong and González-Gómez (2021) analysed the evolution of the attitudes of pre-service teachers’ (PSTs) toward mathematics learning when ABN Method was followed in a flipped classroom. It was conducted in a general mathematics course, Primary Education bachelor’s degree during the course of 2019/2020. A total of 143 students participated in the study (230 students enrolled), with a pre- and post-test survey questionnaire. The results demonstrated that the attitudes (own beliefs and conceptions (OB), positive (ATP), and negative (ATN) attitudes) of PSTs improved positively after completing the flipped-ABN method toward mathematics learning. All questions had a significant difference that showed the influence of the flipped-OCN method, improving the PSTs attitudes toward mathematics learning (*p* < 0.005). The results concluded that this study allowed to draw a promising tendency about the PSTs’ attitudes toward mathematics learning with the ABN method in the flipped classroom. These outcomes could advance our comprehension of how to help pre-service education for teachers’ enhancement and maturity of positive attitudes about themselves as future teachers.

Finally, other recent studies have tried to understand the characteristics of the method and the psychological profiles characterised by mathematical learning through different teaching procedures, either traditional or ABN (Aragón et al., 2017a,b; Cerda et al., 2018; Canto-López et al., 2019). In general, the results in mathematical performance were favourable for the participants of the ABN groups, generating a cognitive profile characterised by good working memory, which is very necessary for arithmetic development. The previous studies of the alternative methodologies that are being more widely used, show other possibilities of teaching mathematics. It is important to note that despite the fact that both methodologies are improving mathematical learning, the ABN method is restructuring the way of teaching mathematics. ABN learning is based on reasoning development from manipulative resources and a deep practice of the system of numeration. These learning bases encourage the development of mental calculation strategies and are supported by an open algorithm that allows problem solving in a constructive way.

It is difficult to compare the research results of the two methodologies, since the research procedures and objectives used are different. However, the empirical evidence on the Singapore method and the ABN method confirms the need to include innovative and manipulative strategies in the classroom, in a way that improves problem solving and the mathematical competence.

In this sense, the main limitations of this manuscript were found in the search for empirical evidence of innovative methods, because it is a relatively recent field of research and to find quantitative studies has been complicated. In addition, the theoretical basis of the methods described is complex, so another limitation was synthesising the information.

Despite limitations, the significance of the findings is interesting for educational purposes, since this overview synthesises somebases for future research in the area. Furthermore, the innovative methods presented could be a reference for the design of new mathematics teaching strategies. As a conclusion, this manuscript contains a scientific value, since it provides a theoretical framework on which to support future empirical work and could provide a theoretical basis for other research groups. The review contributes to broadening the methodological and cognitive bases of some non-traditional mathematics learning approaches.

Regarding the novelty of this manuscript, it should be noted that it is not an original research manuscript. However, the synthesis and organisation of different innovative mathematics teaching strategies, is an innovative element in itself.

Future research should focus on analysing the cognitive processes involved in mathematical learning, carrying out empirical studies. In addition, it is essential to consider the training of future teachers, so that they can learn about new alternatives for mathematical teaching and the available resources to be able to put it into practice. The training programs for Early Childhood Education and Primary Education teachers should be designed, providing work sequences and resources that can be applied in the classroom. The methodological change in the teaching of mathematics is in process. Gradually it will reach all classrooms, in order to achieve mathematical competence in all students.

## Author Contributions

MCC has contributed in literature review about mathematical learning and alternative methods, writing the original manuscript, designing the written and mental calculation figures, and development of the discussion. MMP has contributed in literature review about mathematical learning and alternative methods, writing the manuscript, designing the written and mental calculation figures, and development of the discussion. JCP has contributed to this work with his experience in Didactic mathematics and alternative methods. CM has contributed to the theoretical framework of the ABN method. CD has contributed to the theoretical framework about the Singapore method. EA has contributed in the theoretical framework about other alternative methods. MG has contributed to the literature review about mathematical learning. All authors contributed to the article and approved the submitted version.

## Conflict of Interest

The authors declare that the research was conducted in the absence of any commercial or financial relationships that could be construed as a potential conflict of interest.

## Publisher’s Note

All claims expressed in this article are solely those of the authors and do not necessarily represent those of their affiliated organizations, or those of the publisher, the editors and the reviewers. Any product that may be evaluated in this article, or claim that may be made by its manufacturer, is not guaranteed or endorsed by the publisher.
